# Synthetic biology 2020–2030: six commercially-available products that are changing our world

**DOI:** 10.1038/s41467-020-20122-2

**Published:** 2020-12-11

**Authors:** Christopher A. Voigt

**Affiliations:** Synthetic Biology Center, Department of Biological Engineering, Massachusetts Institute of Technology, Boston, USA

**Keywords:** Metabolic engineering, Synthetic biology

## Abstract

Synthetic biology will transform how we grow food, what we eat, and where we source materials and medicines. Here I have selected six products that are now on the market, highlighting the underlying technologies and projecting forward to the future that can be expected over the next ten years.

“The time has come for synthetic biologists to develop more real-world applications […] the field has had its hype phase, now it needs to deliver.” So concluded an infamous article in 2010^1^. Early research struggled to design cells and physically build DNA with pre-2010 projects often failing due to uncertainty and variability. Since then, rapid technological advances occurred that are well-reviewed in this series of commentaries^2^. Products from synthetic biology are rapidly permeating society and by 2030, it is highly likely that you will have eaten, worn, used or been treated with one.

While there are many biotechnology, pharmaceutical and agriculture companies, I selected those products that best highlight the application of synthetic biology tools developed 2000–2020 and are available now or by early 2021^2–4^. The first three represent chemicals produced by engineered cells or enzymes (leghemoglobin, sitgaliptin, diamines) that are isolated and purified (Fig. 1). For the second three, the products are the engineered cells themselves (engineered bacteria, CAR-Ts, genome edited soy). The development of these was enabled by advances in metabolic engineering, directed evolution (awarded the 2018 Nobel Prize), automated strain engineering, metagenomic discovery, gene circuit design, and genome editing (awarded the 2020 Nobel Prize)^5,6^.

**Fig. 1:**
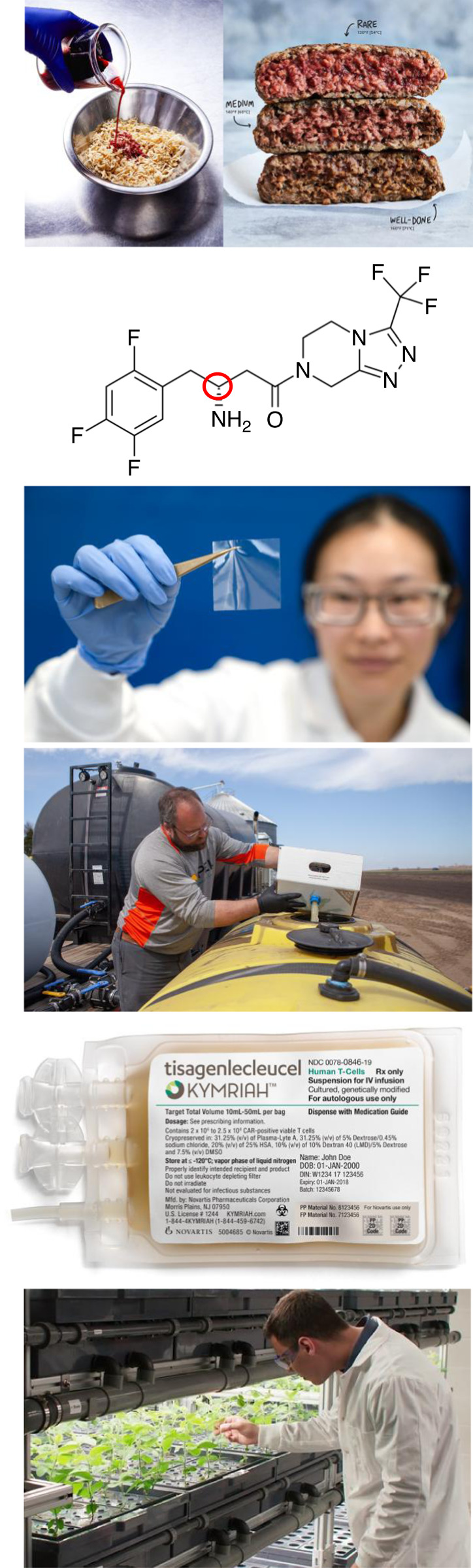
Products from synthetic biology. (Top to bottom) Heme being produced to make an Impossible Burger. Sitgaliptin, with the enzymatically produced stereocenter shown in red. Hyaline transparent and flexible film. ProveN fertilizer being applied to corn. Kymriah, produced by genetically modifying a patient’s T cells. Calyno high-oleic oil from soy. Images were reproduced with permission from Impossible Foods, Zymergen, Pivot Bio, Novartis, and Calyxt, respectively.

## Products from cells

### “Burgers that bleed” by impossible foods

Impossible Foods recognized that blood, specifically the iron-containing heme, is important for the taste and experience of eating a hamburger^7^. Some plant roots “bleed” when cut. The yeast *Pichia pastoris* was engineered to produce soy leghemoglobin, which improves meaty flavors and aromas when added to a plant-based burger^8^. Optimizing the production strain involved DNA synthesis, Gibson Assembly, genetic part libraries and a positive feedback loop for autoinduction. Compared to a beef patty, the Impossible Burger requires 96% less land and 89% fewer greenhouse gases. Worldwide, their products are available in over 30,000 restaurants and 15,000 grocery stores.

Advances in metabolic engineering have simplified the transfer of a pathway from its natural source to a production host, making it possible to move larger pathways to yeast with less effort^9^. This includes computational tools for pathway design, methods to discover and modify enzymes so that they can be ported from plants, precision part libraries to balance their expression, techniques to assemble and integrate large multi-gene pathways and genome editing to redirect carbon flux^9–11^. The palate of food additives that are obtained from engineered yeast is growing rapidly, with products emerging that contain Vitamin E (DSM), Stevia (Amyris and DSM) and milk whey (Perfect Day). Similarly, plant pathways to pharmaceuticals have been moved into yeast, including taxadiene (Taxol precursor), steroids, THC and opiates and efforts are underway to scale-up these processes^9,12,13^. An early landmark achievement to make the anti-malarial Artimisin was taken into production by Sanofi, but was discontinued because the cost was higher than sourcing it from the plant^14,15^. Animal pathways can also be reconstructed; for example, producing shark squalene for vaccines and moth hormones to disrupt an agricultural pest^16,17^. Bioinformatics and high-throughput DNA construction can be used to transfer pathways *en mass* from a plant or microbial community into a production host, for example to screen the human microbiome for putative pharmaceuticals^18,19^. Transferring pathways to fermentation-friendly chasses facilitate access to chemicals present in low quantities in nature and serves as a platform for their enzymatic diversification to produce new molecules.

### Januvia, a diabetes drug from Merck

Januvia (sitgaliptin) increases insulin secretion by inhibiting dipeptidyl peptidase 4. It is the 95th most-prescribed drug with ~10^7^ prescriptions and $1.35 billion in annual sales^20,21^. Stigaliptin has one stereospecific amine that is challenging to manufacture using chemistry alone, requiring heavy metals and high pressures^22^. Starting with a (R)-selective transaminase from *Arthrobacter* sp., the computational design was applied to “open up” the binding pocket to new substrates, followed by rounds directed evolution^6^ to improve its activity under manufacturing conditions. The final enzyme has 27 amino acid substitutions and can achieve >99.95% enantiomeric excess. This approach has also been applied to manufacture the HIV antiviral islatravir for Phase 2 clinical trials using a cascade of five enzymes, all of which are the products of directed evolution^23^. The starting compounds for islatravir and sitgaliptin are highly fluorinated or have an alkyne group that would be difficult to produce using enzymes, requiring chemical steps in manufacturing.

Molecules made by biology can be the envy of chemists, with long retrosynthetic routes to their creation populating the tomes of synthetic organic chemistry^24^. One is tempted to declare biology the ultimate chemist and point to a future where all chemicals are produced by enzymes in cells. This is, however, not accurate. All of the chemistry performed by the natural world can be captured with ~250 reactions, whereas there are 60,000+ in the chemistry literature^12^. Biology is able to build complex-looking structures with repetitious reactions performed on highly functionalized molecules by enzymes with precise specificity and regioselectivity. The illusion of complexity comes from the difficult and circumvent chemical routes required to recreate the molecule. New chemical spaces will be accessible when the power of both chemistry and biology are seamlessly fused, rather than looking to either alone. Enzymes can be incorporated into retrosynthetic routes by mining them from databases using DNA synthesis and screening and controlling their specificity using evolutionary and computational methods^25–28^. Balancing the constraints of chemical-biological routes “by hand” will be near-impossible, thus requiring design software similar to that which has recently revolutionized chemical retrosynthesis^29,30^. Efficiently manufacturing these compounds will require innovations in determining when steps should be performed in a living chasses versus a cell-free system and innovative modular reactor designs^12,30,31^. The formal marriage of chemistry and biochemistry will radically impact everything from medicines to consumer goods and agrochemicals.

### Hyaline, a thin film for electronics by Zymergen

Zymergen’s hyaline is a polyimide film made from bio-sourced monomers^32,33^. Polyimides, most famously Kapton, are associated with being thermally/chemically stable with superior mechanical properties, but normally they have a coloration that prohibits applications requiring transparency^33^. The hyaline family of films are clear, flexible, and mechanically robust making them suitable for flexible electronics (e.g., foldable smartphones and wearable electronics), examples of which will be appearing in products in early 2021^34^. The films are made from diamine monomers produced by engineered organisms that were optimized using a suite of robotics to build millions of strains in parallel, with artificial intelligence learning from the failures to design the next round of strains^33,35,36^. These foundries are emerging globally and accelerating the pace of synthetic biology projects^37^.

Materials have long been sourced from biological sources, but it remains difficult to genetically reprogram the cells to make a new material by design^38–41^. The first materials produced by synthetic biology were small carbon-based monomers that are green drop-in replacements for petroleum products, such as ethanol (Lanzatech/Total), propanediol (DuPont) or butanediol (Genomatica)^3,42–44^. Protein-based materials offer the ability to genetically program the order of monomers in a polymer^45^. Spider silk and related proteins have been produced in fermentations using recombinant cells and prototypes have been announced, such as the Moon Parka by Spiber/North Face^46^, but manufacturing challenges appear to have slowed large-scale product releases. They also simplify the design of new materials, such as elastin-silk chimeras that combine their thermal responsiveness and tensile strength^45,47^. Attempts to port this capability to other polymers, such as polyesters, are being made by engineering the ribosome^48^. Further, biology can control a polymer’s optical or electronic properties by folding it into a nanostructure; for example, the shape of melanin dictates whether it is an UV protectant, luminescent pigment (birds-of-paradise) or photovoltaic cell (wasps)^49,50^. New computational tools are available to build nanostructures of a defined shape out of DNA or proteins^48,51,52^. Biology is not limited to carbon and can build inorganic materials from at least 55 elements, including rare earth and uranium^53^. Using redox enzymes and engineered phage, inorganic nanomaterials have been made for ultralight batteries, catalysts, solar cells, and optics^53,54^.

## Cells as products

### PROVEN, a biological nitrogen fertilizer for corn by Pivot Bio

Farmers must add nitrogen to crops to obtain high yields, most of which is produced using an industrial chemical process that consumes 1–2% of global energy^55^. Bacteria that fix nitrogen from air are used as biological nitrogen fertilizers, but they are not compatible with cereal crops (corn, wheat, rice). Pivot Bio has created the first biological fertilizer for corn based on a γ-proteobacterium (KV137) that associates with corn roots and has the necessary genes to fix nitrogen. However, the genes are off when most needed, so synthetic biology was used to turn the genes on, which guided the remodeling of the KV137 genome^56^. This bacterium is the active ingredient of the liquid fertilizer PROVEN that reduces the need for chemical fertilizer by 25 lbs/acre while increasing yields by 5.8 bushels^57^. Unlike chemical fertilizer, rain does not leach the nitrogen into groundwater, a major source of pollution, or get released into the atmosphere as the powerful greenhouse gas N_2_O. In 2020, PROVEN was used on 250,000 acres, to be expanded to millions in 2021.

Soil, water, and animals harbor complex microbial communities into which there is the potential to add beneficial functions, or remove harmful ones, from these ecosystems^58–60^. Doing this by introducing a new strain can be challenging as the microbiome environment is poorly characterized, dynamic and ecologically rich^61^. The best chasses to deliver a new function are from the target environment, as their ability to prosper in this context is non-trivially encoded in their genome; for example, *Pseudomonas simiae* requires 115 genes to optimally colonize a root^62^. There are many potential chasses from which to choose: microbiomes can be occupied by thousands of species and tools to genetically modify undomesticated bacteria have improved^58,63^. Advances in microfluidics, transformation, and genome editing make it easier to culture species and insert large DNA fragments. Principles from control theory can be applied to create genetic systems that do not require extensive re-tuning in a new chassis (“virtual machines”)^64^. Using these techniques, engineered plant-associated bacteria are being tested to increase crop yields, protect against pests, and increase the range of climates and soil conditions tolerated^62^. More broadly, engineered probiotics can vaccinate chickens, protect honey bees against mites, stop malaria from surviving in mosquitos and as human treatments for infections, inflammation, metabolic disorders, and obesity^65,66^.

### Kymriah, a treatment for B-cell acute lymphoblastic leukemia by Novartis

The therapeutic use of engineered living cells has been described as the “3rd pillar of medicine,” following the era of biologics^67^. Kymriah (Tisagenlecleucel) is the first such therapy to afford FDA approval^68^. CAR-T cells are manufactured by isolating the patient’s T cells, genetically modifying them to express a chimeric antigen receptor (CAR) and reintroducing them into a patient, where they can persist for years, even decades^69,70^. Tisagenlecleucel expresses a fusion between an antibody that targets the CD19 antigen on a cancer cell that is introduced into the patient’s T-cells using a lentivirus^71^. The results are stunning, with an 83% remission rate in patients with relapsed or refractory disease^68^. Kymyriah and the similar Yescarta (Gilead), will together generate ~$1 billion in sales this year^72^. As of summer 2020, there are 671 CAR-T therapies in trials, most targeting blood cancers, but there is an increasing number to treat solid tumors, autoimmune disorders (e.g., multiple sclerosis), and viral infection (e.g., HIV)^70,73^.

Building effective living therapies will require mastery over the design of synthetic regulatory networks (“genetic circuits”)^74^. Genetic circuit design in mammalian cells is being applied to overcome the limitations of the first CAR-T generation^69,74^. Targeting a single cancer antigen can lead to off-target toxicity, such as the long-term depletion of healthy B-cells, and resistance emerges if the antigen mutates^74^. To address these issues, genetic circuits have been designed that integrate information from multiple sensors: AND gates increase specificity and OR gates prevent resistance^69,70^. CAR-T activity in time and space can be controlled using sensors for small molecules that can be administered as drugs or that react to the tumor environment. Safety switches have been designed to trigger rapid CAR-T depletion in case the patient develops cytokine release syndrome, a common and potentially life-threatening side effect^70^. Genetic circuits are sensitive to changes in expression and this leads to variable responses when they are integrated randomly into the genome using lentiviruses; this is addressed by using genome editing to insert into a single “landing pad”^70^. Beyond T cells, genetic circuits and genome editing are critical techniques for controlling where and when therapeutic living cells are active, whether they be patient-derived immune cells or engineered bacteria (e.g., Synlogic’s therapy for phenylketonuria using engineered *E. coli* in clinical trials)^69,74–76^.

### Calyno, a high-oleic oil from soybeans by Calyxt

Calyno oil is the first product from a genome-edited plant to enter the United States food supply. Soybean oil comprises 90% of seed oil, but it is high in linoleic acid, which is not shelf stable and degrades quickly in the fryer. To reduce food waste, it could be partially hydrogenated, but this leads to unhealthy trans-fats^77,78^. Calyxt edited the soy genome to inactivate two fatty acid desaturase genes that reduce the production of the unstable linoleic acid^77^. The deletions yield oil containing 80% oleic acid, whereas unmodified soy only has 20%. The gene editing was performed using transcription activator-like effector nucleases (TALENs), which can be programmed to cut a target DNA sequence^79^. This results in small deletions and no recombinant DNA, thus simplifying regulatory approval, in contrast to a prior effort to silence the genes using RNAi. Calyno oil was launched in 2019 and the genome edited soy is now grown on ~100,000 acres^77,80,81^.

Genome editing has revolutionized biotechnology, and many products are set to enter the market over the next decade, especially in farming and medicine^81^. TALENs and earlier methods can direct changes to genome locations with high fidelity, but they can be hard to design. CRISPR/Cas9 addresses this issue, where an easily designed guide RNA directs the Cas9 nuclease to its target, leading to the replacement, deletion or insertion of genomic DNA^5,82^. Many products are on the way with 140 genome-edited variants of 36 crops that improve yields and nutrition, defeat infections and pests, and expand the range of conditions^81,83^. A better-tasting mustard green (Pairwise) or yield-improved waxy corn (Corteva) could be the first CRISPR/Cas9-constructed product to enter the food supply in 2021^84,85^. Livestock, poultry, and fish are also being genome edited with 67 examples that include hornless cattle (eliminating physical dehorning), sheep with longer wool, goats that make milk with human whey protein, virus-resistant pigs, and chickens that lay allergen-free eggs^83,86^. Human medical treatments are also being developed with genome editing, including safer and more efficacious CAR-T therapies and delivery methods for gene therapies (e.g., Editas’s inherited blindness therapy in clinical trials) and the DNA recognition machinery is being repurposed for in vitro diagnostics of cancer and pathogens^69,87,88^. The pig genome has been edited to be a better host for human organs, with preclinical trials this year, which could alleviate a global shortage of transplant organs (Qihan Bio/eGenesis)^89^.

## What else does the future hold?

A futurist animation envisioned people flying in bee-copters, trees that grow into houses and squid-like living spaceships^90^. This might be hyperbole, but I also see it as a way to depict the emergence of biology-derived components across society: insect materials in aerospace, the toxic glues in architectural materials replaced with mycelia and NASA turning to synthetic biology to produce food and medicine during long space travel^91–93^. The next decade will see more products that derive their superior performance and affordability from engineered biology. Already, the field is having an impact. The products described here total ~$2 billion in annual sales, and the contribution from non-medical applications will grow steadily^94,95^. We are the cusp of a deluge of new innovations; in 2030, writing a commentary such as this one could require reviewing hundreds, if not thousands, of products.

With population increases and more products being derived from fermentation, sugar will become a less viable feedstock to make consumer goods. Over the next decades, new microbial chasses will need to be developed that can derive carbon from alternative sources, such as plastic waste, or CO_2_ from the atmosphere either directly or by coupling to an inorganic “artificial leaf”^96–99^. Fresh water is also a limited resource that is heavily used in fermentation and halophilic chases could be developed that grow in bioreactors containing ocean water^100^. Cell-free manufacturing offers the potential to reduce the water usage, physical footprint and cellular uncertainty^31,101^.

After 2030, products will shift to systems, where cells are designed to work together or be integrated into non-living materials or electronics^102^. In agriculture, functions could be distributed across the engineered plant and bacteria symbioses designed to interlock and communicate with each other and with UAVs, receiving information and sending signals to control gene expression in response^103,104^. The burger patty of the future may be grown using consortia of bacteria, fungi, and livestock cells, similar to yoghurt or cheese, that work together to build tactile structures and synthesize molecules for nutrition, flavor and fragrance. Architectural materials, reminiscent of Singapore’s living buildings, could be embedded with living engineered cells that provide responsive functions, such as self-healing or to clear air pollution^40,41^. To preserve infrastructure, engineered consortia in paints could prevent ship hull biofouling and reduce pipeline corrosion, or they sprayed on soil to stabilize airfield soils^58,105^. Coupling engineered living cells with electronics facilitates brain-computer interfaces and robots that use living sensors for navigation or to generate energy from their environment^102^. Fully realizing this capability requires design tools that are so reliable that millions of variants do not have to be screened and prototyping strategies that extend beyond titer measurements, that can evaluate performance in simulated real-world environments.
